# The Nurse's Library

**Published:** 1921-03-19

**Authors:** 


### THE NURSE'S LIBRARY.

**A Handbook for Nurses.** By T. K. Watson, M.D. (London: The Scientific Press, Ltd. Sixth edition. 10s. 6d. net.)

This book is too well known to need an introduction to the public, but readers of the sixth edition, which now appears, will find that it has been "entirely re-written and much enlarged." The number of illustrations has been increased from 85 to 196, and new chapters on special diseases have been added. Dr. Watson understands the nurse's mind and her limitations. In this comprehensive volume we have anatomy and physiology, medicine and surgery, pharmacology and the science of nursing so cleverly blended, the chapters following each other in so natural a sequence, that the student nurse is not stunned by the bulk of the task before her, but rather is she led to appreciate the relative value of these great subjects and their importance in her work. The diagrams of splints, appliances, bandaging, and instruments are excellent; also the radiograms of foreign bodies, a bismuth meal, and various fractures. A small point of interest is the mention of serge as a possible material for nurses' dresses. Except in a cold, sunny country, such as Northern Egypt in winter, serge is not to be recommended for nurses' dresses, and when it is used it should be light in colour and frequently washed. Nurses who read this book will find a large amount of varied knowledge and wisdom in a very compact form, paving the way to deeper study.

**Bandages, and Bandaging for Nurses.** By Cordelia M. Cowan, President of the Colorado State Board of Nurse Examiners. (W. B. Saunders Company. Philadelphia, London. 10s. 6d. net.)

This admirable little book, in a tabulated "outline," divides itself into purposes and kinds, and under "kinds" we have the usual trio—roller, handkerchief, and tailed bandages. From thence the outline is subdivided, and multiplied as the sands of the sea, into every possible type of bandage; but the student who may be frightened by the new names has only to turn the pages to find the most helpful and clearly explained plates. There are in all 139 photographs, and in each case the width of the bandage and the material to be used are specified. The chapter on plaster bandages contains many useful hints. It would be inexcusable for a nurse to apply an inadequate bandage with this book beside her.

